# Chemometric Analysis of Fourier Transform Infrared Spectra for the Detection of Cotinine in Fingernails of E-Cigarette Users

**DOI:** 10.3390/molecules31050791

**Published:** 2026-02-27

**Authors:** Yong Gong Yu, Putera Danial Izzat Kamaruzaman, Shaun Wyrennraj Ganaprakasam, Nurul Ain Abu Bakar, Eddy Saputra Rohmatul Amin, Muhammad Jefri Mohd Yusof

**Affiliations:** 1 School of Graduate Studies, Postgraduate Centre, Management and Science University, University Drive, Off Persiaran Olahraga, Shah Alam 40100, Malaysia; 2 Department of Diagnostic and Allied Health Science, Faculty of Health and Life Sciences, Management and Science University, University Drive, Off Persiaran Olahraga, Shah Alam 40100, Malaysia; 3 Department of Medicine, Faculty of Medicine and Health Sciences, Universiti Putra Malaysia, Serdang 43400, Malaysia; 4 Forensic Science Programme, Centre of Diagnostic, Therapeutics & Investigation (CODTIS), Faculty of Health Sciences, Basement 1, Perpustakaan Tun Seri Lanang, Universiti Kebangsaan Malaysia, Bangi 43600, Malaysia

**Keywords:** biomarker, keratin, multivariate analysis, nicotine, spectroscopy

## Abstract

Nicotine exposure from e-cigarette use remains a growing public health concern, necessitating reliable biomarkers and analytical approaches for long-term exposure assessment. This study aimed to investigate the feasibility of detecting and classifying cotinine, the primary metabolite of nicotine, in fingernails of e-cigarette users using Fourier transform infrared (FTIR) spectroscopy coupled with chemometric analysis. Fingernail samples were collected and extracted from 30 e-cigarette users and 30 non-smokers. Infrared spectra were acquired in attenuated total reflectance mode and analysed using principal component analysis (PCA) and partial least squares discriminant analysis (PLS-DA) for classification and prediction. Distinct spectral features associated with cotinine were observed in smoker samples, particularly an absorption band near 1277 cm^−1^ corresponding to C–N stretching vibrations. Quantitative analysis revealed significantly higher cotinine concentrations in smokers compared with non-smokers (*p* < 0.05, Mann–Whitney U test). Chemometric modelling achieved complete discrimination between groups, with the PLS-DA model demonstrating excellent predictive performance and an area under the receiver operating characteristic (ROC) curve of 1.0. These findings indicate that FTIR spectroscopy combined with chemometric tools provides a rapid and effective approach for cotinine detection in fingernails, supporting its potential application in nicotine exposure assessment.

## 1. Introduction

Electronic cigarettes, commonly termed e-cigarettes, are electronic nicotine delivery systems that generate an aerosol through heating an e-liquid, which is then inhaled by the user. E-liquids are commercially available in multiple formulations, including nicotine-containing and nicotine-free products [1,2]. Although e-cigarettes are frequently positioned as alternatives to combustible cigarettes, concerns persist regarding long-term use, particularly the potential for nicotine dependence and adverse effects linked to nicotine and other chemical constituents present in e-liquid aerosols [3,4]. In addition, nicotine exposure in e-cigarette users can vary substantially due to differences in device characteristics, power settings, e-liquid composition, and puffing topography [5,6,7].

Nicotine is a potent psychoactive compound whose reinforcing properties contribute to dependence. Following inhalation, nicotine is rapidly absorbed through the pulmonary circulation and distributed systemically, with rapid access to the brain that facilitates reinforcement and self-titration of dose by users [8]. The extent of nicotine exposure is influenced by behavioural and physiological factors, including inhalation depth, puff volume, puffing rate, and intensity, which together contribute to inter-individual variability in circulating nicotine levels [9]. Nicotine metabolism is also affected by biological determinants such as genetic variation, sex, hormonal status, and disease, further complicating the use of nicotine itself as a stable exposure marker [10].

Nicotine is metabolised into several metabolites, of which cotinine is the predominant product, accounting for a substantial proportion of nicotine biotransformation [9,11]. Because nicotine has a relatively short half-life, its measurement typically reflects recent exposure within a narrow time window [8]. In contrast, cotinine exhibits a longer half-life of approximately 16–18 h and greater stability in biological fluids, making it a widely accepted biomarker for assessing nicotine exposure and distinguishing users from non-users [8,10,12,13].

Conventional determination of nicotine and cotinine has relied primarily on chromatographic and immunoassay-based techniques using matrices such as blood, urine, and saliva [8,12]. Although these approaches are analytically robust, they commonly involve extensive sample preparation and can be resource-intensive, with associated chemical consumption and waste generation [14]. Moreover, biological fluids predominantly capture short-term exposure dynamics, which may not be optimal when the objective is retrospective monitoring of nicotine intake. In this context, keratinised matrices such as hair and nails offer advantages for longer-term exposure assessment. Keratin structures can incorporate xenobiotics and metabolites over time, thereby supporting retrospective exposure evaluation and complementing self-report data, which may be incomplete or subject to bias [12,15]. Prior work has reported measurable nicotine and cotinine signals in keratinised specimens, including hair and nails, across active users and individuals with second-hand exposure, highlighting their utility for exposure assessment beyond immediate intake [16,17,18,19].

Evidence from biomonitoring studies highlights the ability of cotinine to reflect nicotine exposure across product types. Elevated cotinine levels have been reported in e-cigarette users relative to non-users in plasma [20], urine [21], and saliva [22], although variability exists depending on usage pattern, product characteristics, and study design. Comparative analyses have shown higher levels of nicotine metabolites among combustible cigarette smokers, followed by dual users and e-cigarette users, with non-users showing the lowest levels, supporting the role of metabolite profiling in exposure stratification [23]. Some studies report overlapping cotinine levels between smokers and e-cigarette users, indicating that classification based solely on concentration thresholds may be unreliable without careful statistical modelling and consideration of exposure sources [24,25]. This is consistent with broader observations that nicotine exposure may occur not only through direct use, but also through passive routes, including second-hand and third-hand exposure and contact with leaked e-liquid, which can confound categorical assignment based on self-reported status alone [26].

Vibrational spectroscopy provides an alternative analytical route for rapid and minimally destructive characterisation of complex samples. Fourier transform infrared (FTIR) spectroscopy has been used across forensic and related analytical domains, including the examination of trace evidence and biological materials, due to its capability to provide molecular-level information with minimal sample requirements and rapid acquisition [27,28,29]. Nevertheless, spectral overlap, matrix contributions, and limited sensitivity for low-abundance targets can complicate interpretation in biological matrices, particularly when the analyte of interest is present at trace levels [30,31]. These considerations make multivariate analysis essential for extracting informative patterns from high-dimensional spectral data.

Meanwhile, chemometrics offers a rigorous statistical framework for interpreting complex spectral datasets by identifying latent structures, quantifying variation, and supporting classification and prediction [32]. Unsupervised methods such as principal component analysis and hierarchical clustering can be used to explore intrinsic sample structure and detect patterns without imposing class labels, whereas supervised methods, including partial least squares discriminant analysis, can be used to develop predictive models that optimise separation and classification performance when class information is available [33]. In vibrational spectroscopy, chemometric modelling is commonly combined with appropriate preprocessing to reduce unwanted variability linked to instrument effects, scattering, and matrix heterogeneity [30]. Prior work across food authenticity and forensic-relevant applications has shown that FTIR coupled with chemometrics can provide discrimination between sample classes and support classification of unknowns in complex matrices [34,35,36]. Related studies have also demonstrated the practicality of applying FTIR and chemometric models to keratinised matrices for exposure classification, including long-term alcohol consumption and other drug-related applications [37,38].

Despite these advances, the combined use of FTIR spectroscopy and chemometric modelling for the detection and classification of cotinine in fingernails among e-cigarette users remains underexplored. Existing literature on nicotine exposure classification has frequently relied on concentration-based comparisons without a consistent and statistically supported approach for defining boundaries between user groups, particularly when passive exposure is plausible [24,25,26]. Given that nicotine delivery from e-cigarettes varies by device characteristics and user behaviour, and may be influenced by evolving product designs, robust analytical approaches that integrate molecular fingerprints with multivariate modelling are needed [5,6].

Therefore, this study evaluates the feasibility of using FTIR spectroscopy coupled with chemometric analysis to detect cotinine in fingernails and classify e-cigarette users and non-users based on spectral patterns. The approach integrates multivariate modelling to support classification in a keratinised matrix, with the broader objective of contributing a rapid, cost-effective, and statistically grounded framework for nicotine exposure assessment.

## 2. Results

### 2.1. FTIR Detection of Cotinine in Fingernails

FTIR spectra obtained from fingernail extracts of non-smokers and e-cigarette users were examined for the presence of absorption bands associated with cotinine.

#### 2.1.1. Non-Smokers

**Figure 1:**
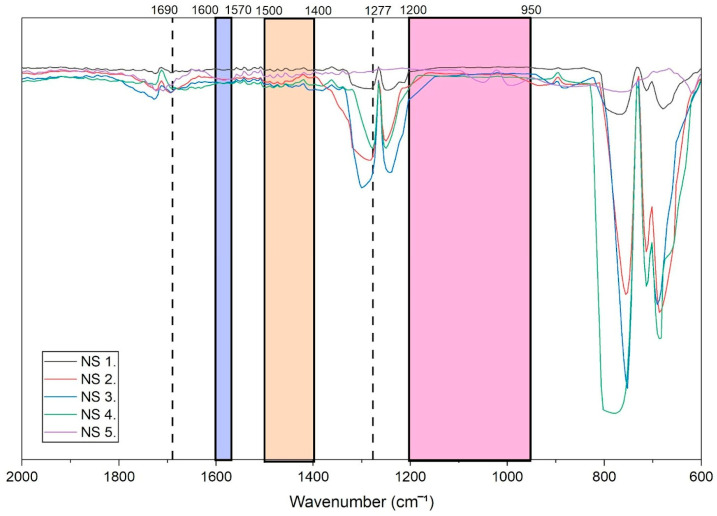
Baseline-corrected FTIR spectrum of fingernail extract obtained from a representative non-smoker. Spectra were pre-processed prior to peak integration and chemometric analysis.

The FTIR spectra of fingernail extracts obtained from non-smokers are presented in Figure 1, where representative spectra are shown to illustrate the spectral features observed in the absence of active nicotine exposure. Overall, the non-smoker spectra exhibited relatively flat and featureless profiles within the spectral regions associated with cotinine when compared with those of e-cigarette users.

Across the region of 950–1200 cm^−1^, which is commonly associated with in-plane C–H and N–H bending vibrations of cotinine-related structures, no pronounced absorption features were observed in the majority of non-smoker samples. Similarly, the region between 1400 and 1500 cm^−1^ showed minimal spectral response, indicating the absence of detectable bending vibrations attributable to CH_3_ and CH_2_ groups linked to cotinine.

A weak absorption feature at approximately 1277 cm^−1^ was observed in some of the non-smoker samples. However, the intensity of this band was markedly lower than that observed in smoker spectra and was not consistently present across the non-smoker group. No distinct absorption features were evident within the regions corresponding to 1570–1600 cm^−1^ or at 1690 cm^−1^. Collectively, the spectral profiles of non-smokers indicate an absence of characteristic cotinine-associated infrared signatures under the conditions employed in this study.

#### 2.1.2. Smokers

**Figure 2:**
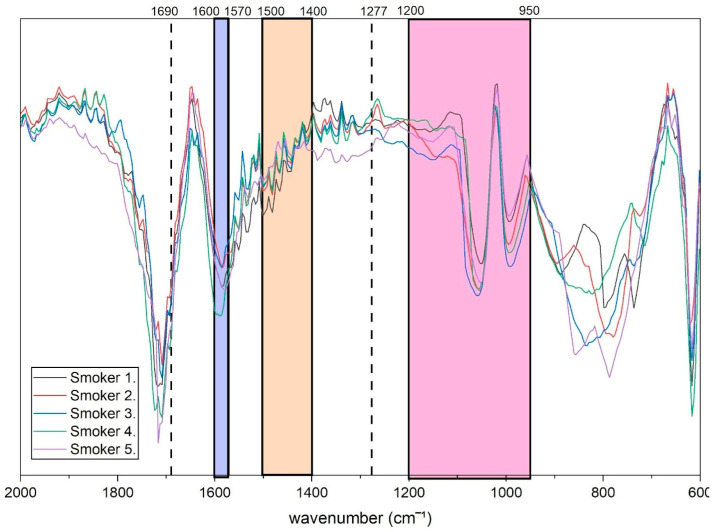
Baseline-corrected FTIR spectrum of fingernail extract obtained from a representative e-cigarette user. Spectra were pre-processed prior to quantitative and multivariate analyses.

The FTIR spectra of fingernail extracts obtained from e-cigarette users are presented in Figure 2, where representative spectra are shown to illustrate the characteristic absorption features associated with cotinine. Compared with the non-smoker group, the spectra of smokers exhibited distinct absorption responses within spectral regions previously reported for cotinine. Notably, consistent absorption features were observed in the range of 950–1200 cm^−1^, which corresponds to in-plane C–H and N–H bending vibrations within the cotinine molecular structure [39]. These features were reproducible across the majority of smoker samples. A prominent absorption band was observed at approximately 1277 cm^−1^ in nearly all samples. In addition, several smoker spectra displayed absorption features within the region of 1400–1500 cm^−1^, which has been associated with bending vibrations of CH_3_ and CH_2_ groups related to cotinine-containing structures.

### 2.2. Comparative FTIR Spectral Analysis Between Smokers and Non-Smokers

**Figure 3:**
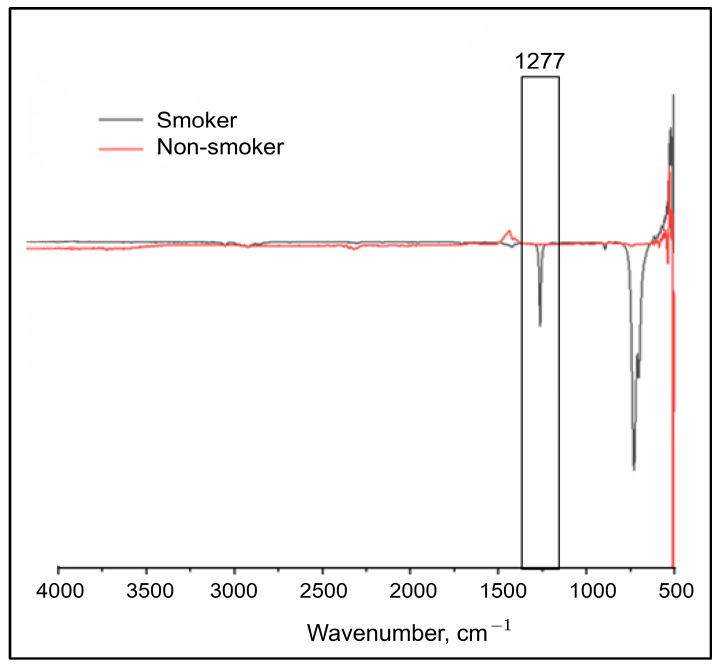
Comparative baseline-corrected FTIR spectra of fingernail extracts from an e-cigarette user (black) and a non-smoker (red), highlighting the absorption band at 1277 cm^−1^ used for cotinine quantification.

A comparative evaluation of the FTIR spectra obtained from smokers and non-smokers was conducted to identify distinguishing spectral features and to support cotinine quantification. Representative overlaid spectra for both groups are presented in Figure 3.

The most pronounced difference between the two groups was observed at approximately 1277 cm^−1^, where smoker samples exhibited a markedly higher absorption intensity compared with non-smokers. This difference was consistently observed across the analysed samples, supporting the selection of the 1277 cm^−1^ band for peak integration and subsequent estimation of cotinine concentration.

The infrared absorption region spanning 1180–1280 cm^−1^ has been associated with C–N stretching vibrations in unsaturated amine structures [40]. In the present study, the absorption band centred at approximately 1277 cm^−1^ corresponds to the C–N stretching vibration characteristic of cotinine. Cotinine contains a pyridine ring linked to a pyrrolidine moiety, and conjugation between these structural units contributes to partial double-bond character within the C–N bond.

The presence and spectral position of the absorption band observed in this study are consistent with previous reports, in which C–N stretching vibrations of cotinine were identified near 1277 cm^−1^ in serum samples [39]. The agreement between the present findings and established literature supports the reliability of this spectral marker for cotinine detection in fingernail matrices. 

### 2.3. Statistical Analysis of Cotinine Detection

Cotinine was selected as the biomarker of nicotine exposure and quantified by integrating the peak area of the absorption band at 1277 cm^−1^ using a previously validated calibration model [41]. The model demonstrated excellent linearity (R^2^ = 0.998), with high precision (relative standard deviation, RSD = 0.86%, *n* = 3) and satisfactory accuracy (recovery = 95.5%). Precision was evaluated through triplicate measurements, while accuracy was determined by recovery analysis of cotinine-spiked samples. The quantified cotinine concentrations in fingernail samples from smokers and non-smokers are presented in Table 1.

The mean cotinine concentration in the smoker group was 463.99 ± 519.13 µg/kg, whereas the non-smoker group exhibited a substantially lower mean concentration of 6.58 ± 23.07 µg/kg. The median concentration in the smoker group was 227.65 µg/kg with an interquartile range (IQR) of 568.24 µg/kg. In contrast, the non-smoker group showed a median of 0.00 µg/kg and an IQR of 0.00 µg/kg.

Values reported as “ud” indicate concentrations below the analytical limit of detection. For statistical analysis, these undetected values were assigned a value of 0.00 µg/kg. Therefore, the reported 0.00 values represent concentrations below the detection limit rather than confirmed absolute absence of cotinine.

The wide dispersion observed in the smoker group, reflected by the large standard deviation and IQR, indicates substantial inter-individual variability in cotinine accumulation among e-cigarette users.

Next, normality of cotinine concentration data was assessed using the Shapiro–Wilk test (Table 2). The results demonstrated significant deviation from normal distribution in both the smoker group (W = 0.785, *p* < 0.001) and the non-smoker group (W = 0.327, *p* < 0.001), indicating highly skewed data. Therefore, parametric testing was deemed inappropriate.

A non-parametric Mann–Whitney U test was subsequently performed to compare cotinine concentrations between the two groups. The analysis revealed a statistically significant difference between smokers and non-smokers (U = 858.0, z = 6.404, *p* < 0.001; *n* = 60). Smokers exhibited substantially higher cotinine concentrations (mean rank = 44.10) than non-smokers (mean rank = 16.90). The magnitude of the difference was large (r = 0.83; r = |z|/√N), indicating a strong separation in cotinine concentration distributions between the two groups.

### 2.4. Chemometric Analysis of FTIR Spectra

Chemometric analysis was applied to classify fingernail samples from smokers and non-smokers based on their FTIR spectral data. Prior to multivariate analysis, spectral preprocessing was performed to improve data quality and ensure consistency across samples. Baseline correction was applied, and the spectral region from 800 to 4000 cm^−1^ was selected for subsequent analysis. Two chemometric techniques were employed, namely principal component analysis (PCA) and partial least squares discriminant analysis (PLS-DA).

#### 2.4.1. PCA

PCA was used as an unsupervised exploratory method to examine clustering patterns within the dataset prior to supervised classification. PCA was performed on the preprocessed FTIR spectra to evaluate the distribution of smokers and non-smokers in reduced dimensional space. The eigenvalues and variance explained by the principal components are summarised in Table 3.

**Figure 4:**
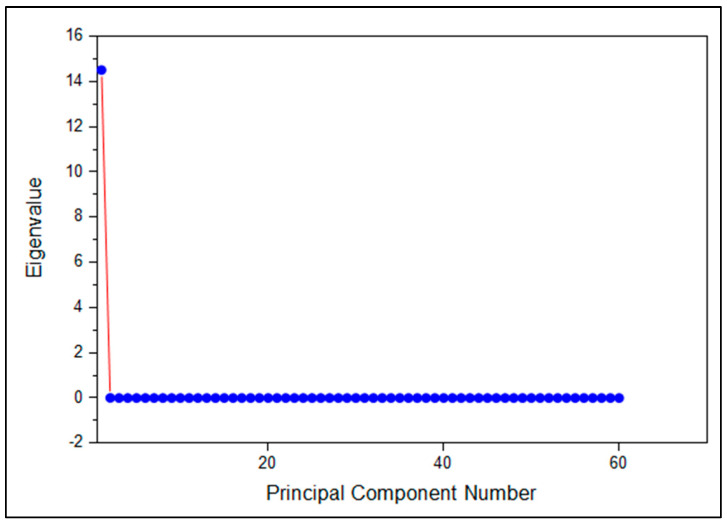
Scree plot showing the eigenvalues of principal components derived from FTIR spectral data of fingernail samples.

The scree plot shown in Figure 4 illustrates the distribution of eigenvalues across principal components. The first principal component (PC1) accounted for approximately 99.96% of the total variance, while subsequent components contributed negligibly to the overall variance. Based on the eigenvalue criterion and scree plot analysis, PC1 was identified as the dominant component.

**Figure 5:**
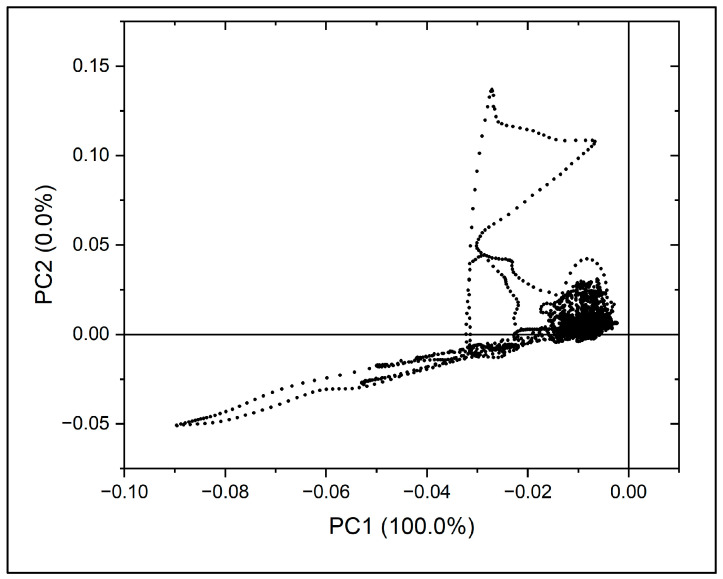
Loading plot of the first two principal components (PC1 and PC2) obtained from FTIR spectral data, displaying the contribution of wavenumbers to variance.

The loading plot of PC1 versus PC2 is presented in Figure 5 to display the contribution of individual wavenumbers to the principal components. Most variables were clustered near the origin, indicating minimal influence on variance, while selected wavenumbers exhibited positive or negative loadings, reflecting their contribution to spectral differentiation.

**Figure 6:**
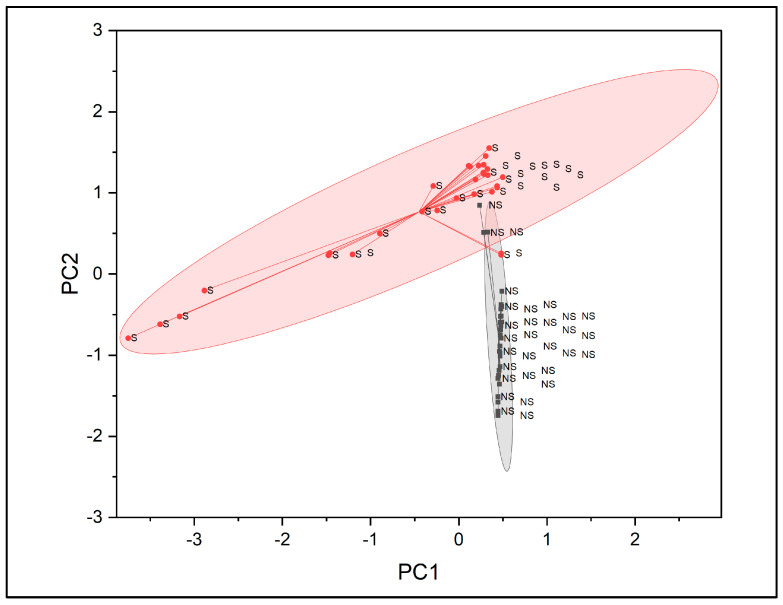
PCA score plot based on PC1 and PC2, showing clustering of fingernail samples from smokers and non-smokers [S = smoker, NS = non-smoker].

Sample scores extracted from PC1 and PC2 were used to visualise clustering patterns, as shown in Figure 6. Two distinct clusters corresponding to smokers and non-smokers were observed, with minimal overlap between groups. The smoker cluster displayed greater dispersion compared with the non-smoker cluster, indicating higher variability among smoker samples. Non-smoker samples formed a more compact cluster, reflecting greater spectral homogeneity.

#### 2.4.2. PLS-DA

PLS-DA was applied as a supervised chemometric method to further evaluate classification performance based on FTIR spectral data. PLS-DA was employed to model the relationship between the predictor matrix (X, FTIR spectra) and the response variable (Y, class membership: smoker or non-smoker).

Model optimisation was performed using cross-validation, and the root mean predicted residual sum of squares (PRESS) values for successive latent variables are summarised in Table 4. The lowest PRESS value was obtained using two latent variables, indicating that this configuration provided the optimal balance between model complexity and predictive performance.

The proportion of variance explained by the PLS-DA model is presented in Table 5. The first latent variable accounted for 99.95% of the variance in X, while the first two latent variables together explained 87.12% of the variance in Y. Additional latent variables contributed negligibly to variance explanation.

**Figure 7:**
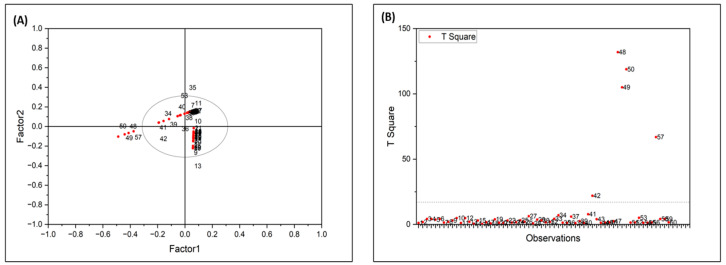
(**A**) Score plot based on the first two latent variables (LV1 and LV2) derived from FTIR spectral data of fingernail samples. (**B**) Hotelling’s T^2^ plot illustrating sample leverage relative to the PLS-DA model.

The PLS-DA score plot and Hotelling’s T^2^ plot for the first two latent variables are shown in Figure 7A and Figure 7B, respectively. The score plot illustrates the overall distribution of samples within the latent variable space, while the Hotelling’s T^2^ plot highlights samples with higher leverage relative to the model centre. Samples positioned away from the central cluster indicate stronger spectral variation and may reflect higher cotinine concentrations. The score plot was primarily used to assess sample dispersion and potential outliers rather than direct visual class separation.

**Figure 8:**
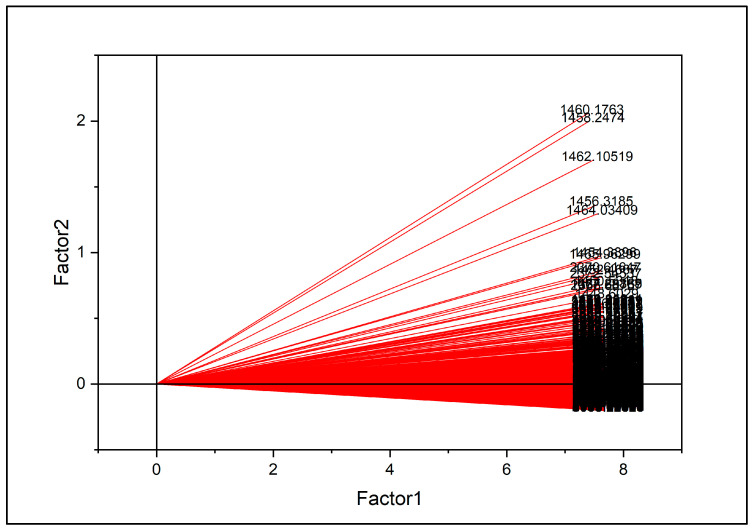
Loading plot for the first two latent variables (LV1 and LV2), showing the contribution of FTIR wavenumbers to model construction.

The loading plot for the first two latent variables is shown in Figure 8, illustrating the contribution of individual wavenumbers to the PLS-DA model. Wavenumbers located farther from the origin exhibited greater influence on latent variable construction.

**Figure 9:**
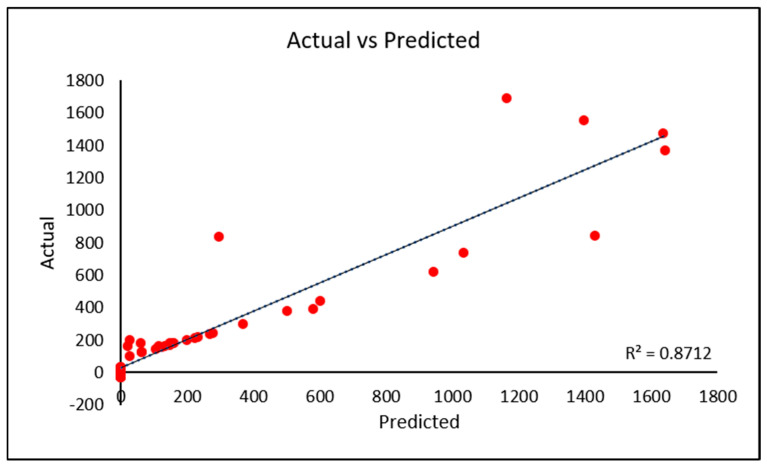
Plot of predicted versus measured cotinine concentrations obtained from the regression model based on FTIR spectral data.

Model performance for quantitative prediction is illustrated in Figure 9, which compares predicted and measured cotinine concentrations. The coefficient of determination (R^2^) for the model was 0.8712, indicating strong agreement between predicted and actual values.

Next, classification results generated by the PLS-DA model are presented in Table 6. The model achieved complete separation of smoker and non-smoker classes in the training set, with all smoker samples correctly classified. Three non-smoker samples were classified as smokers, and two smoker samples were classified as non-smokers.

To further assess the efficacy of the class separation model, several validation analyses were performed using the latent variable scores derived from the PLS model. The univariate ANOVA results for the discriminant functions are presented in Table 7, showing statistically significant contributions from both latent variables (*p* < 0.05).

The eigenvalue and canonical correlation associated with the discriminant function are shown in Table 8. A single discriminant function was obtained with an eigenvalue of 2.14924, accounting for 100% of the between-group variance. The corresponding canonical correlation was 0.82611, reflecting a strong association between the linear combination of the latent variables and class membership.

The effectiveness of the discriminant function was further assessed using Wilks’ lambda test, as shown in Table 9. A Wilks’ lambda value of 0.31754 was obtained, with a statistically significant chi-square result (*p* < 0.0001), indicating significant separation between smoker and non-smoker groups.

**Figure 10:**
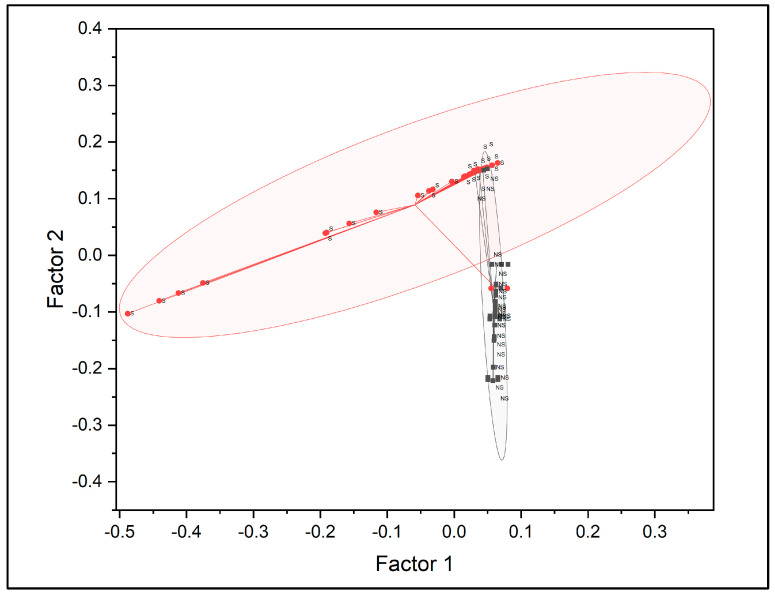
Discriminant score plot derived from PLS latent variables, illustrating classification of fingernail samples from smokers and non-smokers [S = smoker, NS = non-smoker].

Lastly, the discriminant score plot derived from the two latent variables is presented in Figure 10, illustrating the distribution of samples according to class membership.

#### 2.4.3. Performance Evaluation of the PLS-DA Model

A PLS-DA model was constructed using FTIR spectral data from 30 smokers and 30 non-smokers. The model yielded a predictive coefficient (R^2^) of 0.8712 for cotinine concentration. To evaluate classification performance, a set of 10 samples not included in model training was treated as an external test set. These samples were used as blind matrices to assess the predictive capability of the model.

The predicted class assignments for the external test samples are presented in Table 10, together with their actual class labels. All test samples were correctly classified by the PLS-DA model, corresponding to a classification accuracy of 100% for the external validation set.

**Figure 11:**
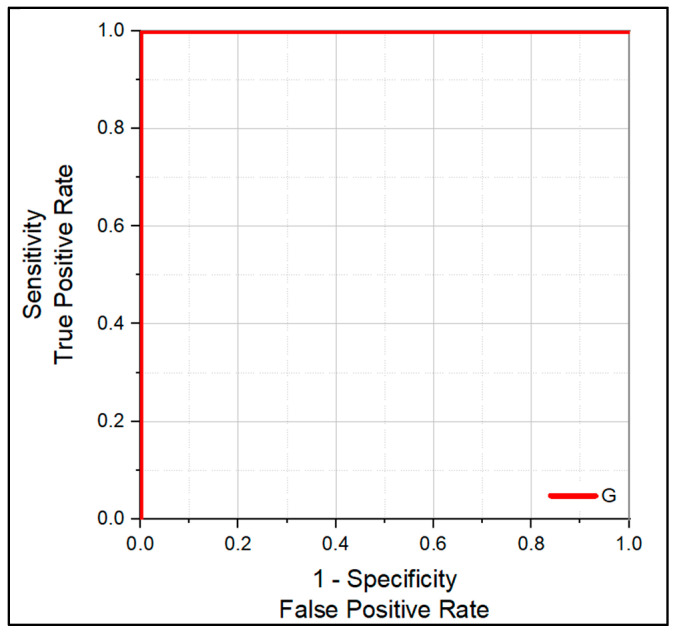
ROC curve for the PLS-DA classification model.

Additionally, a receiver operating characteristic (ROC) curve was generated to evaluate the classification performance of the PLS-DA model for distinguishing smokers from non-smokers. The ROC curve, shown in Figure 11, was constructed using specificity on the x-axis and sensitivity on the y-axis. Model performance was assessed by calculating the area under the curve (AUC).

The AUC values obtained from the ROC analysis are tabulated in Table 11. The PLS-DA model achieved an AUC value of 1.0, corresponding to 100% sensitivity and 100% specificity for the classification of smoker and non-smoker samples.

## 3. Discussion

### 3.1. Detection of Cotinine in Fingernails Using FTIR Spectroscopy

The detection of cotinine in fingernail samples using FTIR spectroscopy was achieved through the identification of characteristic absorption bands associated with the molecular structure of cotinine. In particular, the absorption band observed in the 1180–1280 cm^−1^ region, with a prominent peak centred near 1266–1277 cm^−1^, corresponds to C–N stretching vibrations of unsaturated amine structures [39,40,41]. This spectral feature was consistently present in samples from e-cigarette users and largely absent in non-smokers, supporting its suitability as a marker for cotinine detection in keratinised matrices.

The observed spectral assignments are in agreement with earlier studies reporting C–N stretching vibrations of cotinine at approximately 1277 cm^−1^ in serum and other biological samples [39]. The molecular structure of cotinine, which comprises a pyridine ring linked to a pyrrolidine moiety, contributes to partial double-bond character in the C–N bond through conjugation effects, thereby enhancing the infrared activity of this vibrational mode [41,42]. The reproducibility of this absorption feature across smoker samples indicates that FTIR spectroscopy provides sufficient sensitivity to detect cotinine in fingernails following appropriate extraction.

A comparable study has previously reported the detection of cotinine in hair matrices using FTIR spectroscopy. That investigation represented preliminary research into the detection of cotinine in keratinised samples using a spectroscopic approach. The extraction procedure adopted in the present study was based on this earlier work, with optimisation of extraction parameters specifically tailored for fingernail matrices [41,43]. To our knowledge, limited studies have investigated cotinine detection in fingernails using FTIR spectroscopy. Therefore, the present work contributes to improving analytical sensitivity for cotinine detection in fingernail samples using this technique. In contrast, comprehensive studies quantifying cotinine in hair and nail matrices have primarily relied on chromatographic methods. Keratinised samples analysed by chromatography are typically subjected to extensive pre-treatment procedures involving various extraction techniques, particularly solid-phase microextraction (SPME). SPME is widely recognised for its ability to pre-concentrate analytes, thereby enhancing sensitivity and specificity, with reported LOD ranging from 0.13 to 200 pg/mL and limits of quantification (LOQ) ranging from 50 to 500 pg/mL [44,45,46].

### 3.2. Quantitative Variability of Cotinine Among E-Cigarette Users

Quantitative analysis revealed substantial variability in cotinine concentrations among individuals within the smoker group, with measured values ranging from non-detectable levels to concentrations exceeding 1600 µg/kg. Such variability is consistent with previous biomonitoring studies using keratinised tissues and reflects the complex interplay of behavioural, physiological, and exposure-related factors influencing nicotine uptake and metabolism [9,47].

Differences in e-cigarette usage patterns, including frequency of use, puff duration, and device characteristics, are likely contributors to the observed variability. Additionally, individual metabolic differences may influence the conversion of nicotine to cotinine and its subsequent incorporation into nails [48]. In the context of e-cigarette use, leakage of e-liquid during aerosolisation has been reported and may result in unintentional inhalation of higher nicotine doses, potentially explaining elevated cotinine concentrations in certain users [49].

Two smokers exhibited no detectable cotinine despite self-reported e-cigarette use. This finding may be attributed to the consumption of nicotine-free e-liquids, which are widely available in the Malaysian market [50]. This highlights an inherent limitation in exposure assessment studies relying solely on self-reported usage and underscores the value of biomarker-based verification.

### 3.3. Passive Exposure and Cotinine Detection in Non-Smokers

Low levels of cotinine were detected in a small subset of non-smokers, despite the absence of reported active use. This observation is consistent with earlier studies demonstrating that keratinised tissues can capture passive exposure to nicotine-containing aerosols. Avila-Tang et al. [51] reported detectable nicotine levels in toenails of non-smokers exposed to second-hand smoke, while Inukai et al. [52] identified cotinine in hair samples of individuals without direct tobacco use.

Further evidence of passive exposure has been documented in neonatal studies, where nicotine and cotinine were detected in nails of newborns, including those born to non-smoking mothers, suggesting in utero exposure to environmental tobacco smoke [53,54,55]. These findings collectively support the utility of fingernails as a long-term exposure matrix capable of capturing low-level environmental nicotine exposure. The detection of cotinine in non-smokers in the present study is consistent with previous observations and underscores the importance of considering passive exposure when interpreting biomarker data. Moreover, the presence of low levels of cotinine in non-smokers raises concerns regarding involuntary nicotine exposure and highlights its relevance for public health monitoring.

### 3.4. Chemometric Discrimination and Predictive Performance

The integration of chemometric techniques with FTIR spectroscopy significantly enhanced the analytical capability of the proposed approach. Principal component analysis revealed clear clustering between smoker and non-smoker samples, although greater dispersion was observed within the smoker group, reflecting the heterogeneity of cotinine concentrations. This pattern is consistent with the quantitative findings and supports the use of multivariate methods to interpret complex spectral data.

The application of partial least squares discriminant analysis further improved class separation and predictive performance. The PLS-DA model demonstrated strong agreement between predicted and measured cotinine concentrations, with robust classification performance confirmed through external validation and ROC analysis. These findings are consistent with previous reports highlighting the effectiveness of supervised chemometric methods in resolving subtle spectral differences and improving classification accuracy in spectroscopic studies [56,57,58,59,60].

The perfect separation observed between smoker and non-smoker groups in ROC analysis shows the discriminatory power of the selected spectral features when combined with multivariate modelling. Importantly, the correct classification of external test samples suggests that the model is not limited to the training dataset and may be applicable to independent sample sets.

### 3.5. Methodological Considerations and Future Directions

While this study demonstrates the feasibility of FTIR spectroscopy coupled with chemometrics for cotinine detection in fingernails, several limitations warrant consideration. The exclusive use of cotinine as the biomarker was motivated by its longer half-life and reported higher stability in keratinised tissues compared with nicotine [61]. However, previous studies have reported instances where nicotine and cotinine concentrations do not correlate within the same sample [47]. Future work incorporating both biomarkers may provide a more comprehensive understanding of nicotine exposure dynamics.

Additionally, the present study did not investigate associations between cotinine concentrations and detailed usage patterns or physiological variables. Longitudinal studies incorporating structured exposure assessments and repeated sampling may provide further insight into temporal exposure trends and inter-individual variability. Such approaches could also facilitate the establishment of threshold values to distinguish active use from passive exposure. Beyond behavioural factors, methodological aspects warrant further investigation. In particular, storage conditions of fingernail samples and the influence of different anatomical regions of the fingernails on cotinine accumulation should be systematically evaluated in future studies.

### 3.6. Implications for Exposure Assessment and Public Health Monitoring

The increasing prevalence of e-cigarette use and associated second-hand aerosol exposure in occupational, domestic, and public settings underscores the need for reliable exposure assessment tools. Previous studies have demonstrated the utility of hair and nail matrices for differentiating active smokers, passive smokers, and non-exposed individuals based on biomarker concentrations [12,15,17,47,52].

The analytical framework presented in this study offers a rapid, non-destructive, and environmentally sustainable alternative for nicotine exposure monitoring. The combination of FTIR spectroscopy with chemometric analysis has the potential to support large-scale screening and contribute to public health surveillance, forensic investigations, and regulatory assessments related to e-cigarette use.

## 4. Materials and Methods

### 4.1. Study Design

This study employed an analytical cross-sectional design integrating experimental spectroscopy with multivariate statistical analysis. The workflow comprised fingernail sample collection, the extraction of cotinine, FTIR spectroscopic analysis, and subsequent chemometric modelling for classification and prediction. Chemometric techniques were applied to address the complexity of spectral data and enable discrimination between e-cigarette users and non-smokers based on FTIR spectral patterns.

### 4.2. Participant Selection and Ethical Approval

A total of 60 participants were recruited using purposive and snowball sampling strategies within the Klang Valley, Selangor, Malaysia. The study population consisted of 30 e-cigarette users and 30 non-smokers, selected irrespective of sex or ethnicity. To minimise confounding factors affecting nicotine and cotinine incorporation into keratinised tissues, all participants were required to be free from diagnosed medical conditions and not undergoing pharmacological treatment.

Eligibility criteria for e-cigarette users included individuals aged 18 to 40 years with a minimum of five months of continuous e-cigarette use and no prior history of combustible tobacco smoking. Non-smokers were defined as individuals aged 18 to 40 years with no lifetime use of tobacco or e-cigarette products and no regular exposure to second-hand smoke or e-cigarette aerosol within the preceding five months. The minimum exposure duration was selected to correspond with the average growth period required for nail formation from the germinal matrix to the free edge.

Participants were excluded if fingernail samples had undergone cosmetic chemical treatments, such as manicures, or exhibited physical damage or pathological abnormalities. Written informed consent was obtained from all participants prior to sample collection. Ethical approval for the study was granted by the University Ethics Committee of Management and Science University (Approval No. EA-L2-01-FHLS-2024-06-0002).

### 4.3. Fingernail Sample Collection

Fingernail collection procedures were standardised across all participants. Prior to sample collection, participants were instructed to wash their hands thoroughly with soap and water and allow them to air-dry. Fingernail clippings were collected from all ten fingers using clean stainless-steel clippers. Approximately one week of nail growth was collected per participant, yielding sample masses ranging from 10 to 30 mg.

Collected samples were placed individually into labelled polyethylene zip-lock bags and stored at room temperature under dark conditions to minimise potential degradation. Samples from different participants were handled and stored separately to prevent cross-contamination.

### 4.4. Extraction of Cotinine from Fingernails

The extraction of cotinine from fingernail samples followed an optimised protocol previously developed and validated using response surface methodology, as reported by Yu et al. [41]. Briefly, decontaminated fingernail samples were subjected to solvent-assisted digestion and liquid–liquid extraction under optimised conditions to release cotinine from the keratin matrix. Extracts obtained from this procedure were directly used for FTIR spectroscopic analysis in the present study.

### 4.5. FTIR Analysis

FTIR measurements were performed using an FTIR spectrometer (IRAffinity-1, Shimadzu, Kyoto, Japan) equipped with an attenuated total reflectance (ATR) accessory. Spectra were acquired over the wavenumber range of 400 to 4000 cm^−1^ at a spectral resolution of 4 cm^−1^, with 20 scans co-added for each measurement. Background spectra were recorded prior to each sample analysis.

The ATR crystal was cleaned with acetone between measurements to remove residual material and prevent cross-contamination. Spectral acquisition and initial data handling were conducted using LabSolutions IR 2.3 software (Shimadzu).

### 4.6. Spectral Preprocessing

**Figure 12:**
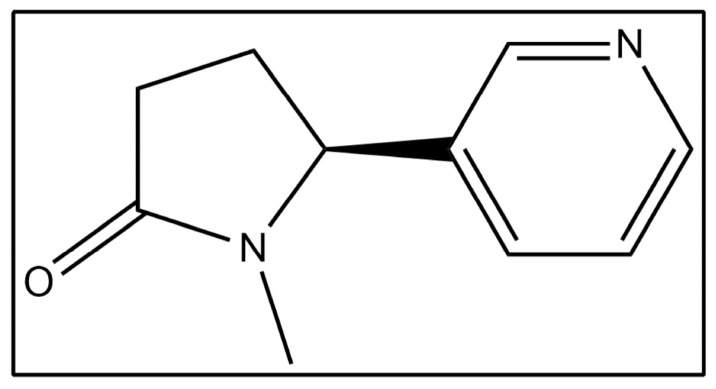
Molecular structure of cotinine.

Spectral preprocessing was performed using OriginPro 2026 software (OriginLab Corporation, Northampton, MA, USA) to improve comparability and analytical robustness. Raw spectra were subjected to baseline correction, normalisation, and smoothing to reduce instrumental variation and background interference. Identification of the characteristic absorption bands associated with cotinine was guided by previously reported infrared spectral assignments, as presented in Table 12, together with the corresponding molecular structure illustrated in Figure 12 [39,41]. All FTIR spectra were subjected to baseline correction prior to peak integration at 1277 cm^−1^ and subsequent chemometric analysis to minimise background variation and improve signal reliability.

### 4.7. Quantification of Cotinine and Statistical Analysis

Cotinine concentrations were estimated by integrating characteristic FTIR absorption bands and expressed as mean ± standard deviation in µg/kg. Statistical comparisons between e-cigarette users and non-smokers were conducted using non-parametric Mann–Whitney U test. Statistical significance was defined at *p* < 0.05. Data analysis was performed using SPSS 26 software (IBM Corp., Armonk, NY, USA).

### 4.8. Chemometric Analysis

#### 4.8.1. Exploratory Analysis

PCA was applied as an unsupervised method to explore intrinsic patterns within the FTIR spectral dataset, assess sample distribution, and identify potential outliers. PCA reduced the dimensionality of the spectral data while retaining the principal sources of variance.

#### 4.8.2. Supervised Classification

PLS-DA was employed as a supervised classification technique to distinguish between e-cigarette users and non-smokers. The method projects spectral variables into latent variables that maximise covariance between predictor variables (FTIR spectra) and class membership. Model performance was evaluated using cross-validation to assess robustness and minimise overfitting.

#### 4.8.3. Model Performance Evaluation

Classification performance was assessed using sensitivity, specificity, and overall accuracy derived from confusion matrix analysis. ROC curves were generated to evaluate model discrimination, and the AUC was calculated as an indicator of classification performance.

### 4.9. Ethical Considerations

All experimental procedures involving human participants were conducted in accordance with the approved ethical protocol. Participant confidentiality was maintained throughout the study, and all data were processed solely for research purposes.

## Figures and Tables

**Table 1:** Individual fingernail cotinine concentrations quantified by FTIR peak integration at 1277 cm^−1^ in e-cigarette users and non-smokers (*n* = 30 per group).

No.	Smokers (µg/kg)	Non-Smokers (µg/kg)
1	103.86	ud
2	61.67	ud
3	62.12	ud
4	1033.82	ud
5	231.75	ud
6	501.00	112.30
7	24.87	58.32
8	366.99	ud
9	942.13	ud
10	601.56	ud
11	1430.71	ud
12	295.07	ud
13	19.91	ud
14	223.56	ud
15	148.14	ud
16	156.42	ud
17	131.15	ud
18	1634.61	ud
19	1395.14	ud
20	1164.47	ud
21	160.22	ud
22	197.84	ud
23	579.93	ud
24	268.20	ud
25	275.55	ud
26	145.61	26.84
27	1639.99	ud
28	0.00	ud
29	0.00	ud
30	123.33	ud
Mean ± SD	463.99 ± 519.13	6.58 ± 23.07
Median	227.65	0.00
Interquartile range (IQR)	568.24	0.00

ud = undetected; values below the limit of detection (LOD). For statistical analysis, undetected values were treated as 0.00 µg/kg.

**Table 2:** Normality test (Shapiro–Wilk) results of smokers and non-smokers groups.

	Statistic	df	Sig.
Smokers	0.785	30	0.000
Non-smokers	0.327	30	0.000

**Table 3:** Eigenvalues and explained variance of principal components derived from FTIR spectral data.

Principal Component	Eigenvalue	Variance Explained (%)	Cumulative Variance (%)
PC1	14.51437	99.96412	99.96412
PC2	0.00521	0.03588	100.00000
PC3	1.54 × 10^−29^	1.06 × 10^−28^	100.00000

**Table 4:** Cross-validation results showing root mean PRESS values for PLS-DA models with increasing numbers of latent variables.

Number of Factors	Root Mean PRESS
0	1.01695
1	0.42483
2	0.40713
3	0.40713

**Table 5:** Percentage of variance explained by the PLS-DA model for predictor (X) and response (Y) variables.

Number of Factors	X Variance Explained (%)	Cumulative X (%)	Y Variance Explained (%)	Cumulative Y (%)
1	99.94901	99.94901	85.75145	85.75145
2	0.05099	100.00	1.36674	87.11819
3	0.00	100.00	1.54 × 10^−9^	87.11819

**Table 6:** Classification results obtained from the PLS-DA model for fingernail samples from smokers and non-smokers.

Actual Class (Sample ID)	Predicted Class	Actual Class (Sample ID)	Predicted Class
NS (1)	NS	S (31)	S
NS (2)	NS	S (32)	S
NS (3)	NS	S (33)	S
NS (4)	NS	S (34)	S
NS (5)	NS	S (35)	S
NS (6)	S	S (36)	S
NS (7)	S	S (37)	S
NS (8)	NS	S (38)	S
NS (9)	NS	S (39)	S
NS (10)	NS	S (40)	S
NS (11)	NS	S (41)	S
NS (12)	NS	S (42)	S
NS (13)	NS	S (43)	S
NS (14)	NS	S (44)	S
NS (15)	NS	S (45)	S
NS (16)	NS	S (46)	S
NS (17)	NS	S (47)	S
NS (18)	NS	S (48)	S
NS (19)	NS	S (49)	S
NS (20)	NS	S (50)	S
NS (21)	NS	S (51)	S
NS (22)	NS	S (52)	S
NS (23)	NS	S (53)	S
NS (24)	NS	S (54)	S
NS (25)	NS	S (55)	S
NS (26)	S	S (56)	S
NS (27)	NS	S (57)	S
NS (28)	NS	S (58)	NS
NS (29)	NS	S (59)	NS
NS (30)	NS	S (60)	S

[S = smoker, NS = non-smoker].

**Table 7:** Univariate ANOVA results for discriminant analysis based on PLS latent variables.

Factor	R-Square	F Value	df	df2	Prob>F
Factor 1	0.20645	15.08947	1	58	2.65533 × 10^−4^
Factor 2	0.47601	52.68944	1	58	<0.0001

**Table 8:** Eigenvalues of discriminant analysis.

	Eigenvalue	Percentage of Variance	Cumulative	Canonical Correlation
1	2.14924	100%	100%	0.82611

**Table 9:** Wilks’ Lambda Test.

	Wilks’ Lambda	Chi-Square	df	Sig.
1 to 1	0.31754	65.38826	2	<0.0001

**Table 10:** Classification results of 10 external test samples predicted using the trained PLS-DA model.

Actual Class (Sample ID)	Predicted Class
NS (1)	NS
NS (2)	NS
S (3)	S
NS (4)	NS
NS (5)	NS
S (6)	S
S (7)	S
NS (8)	NS
NS (9)	NS
S (10)	S

[S = smoker, NS = non-smoker].

**Table 11:** ROC analysis of the PLS-DA model.

Area	Std. Error	Asymptotic Prob	95% LCL	95% UCL
1	0	<0.0001	1	1

**Table 12:** Infrared spectral regions associated with cotinine and their corresponding bond vibration assignments.

Spectral Region, cm^−1^	Bond Vibration Assignment
950–1200	In-plane C–H and N–H bending vibrations
1277	C–N stretching vibrations
1400–1500	Bending vibrations of CH_3_ and CH_2_ groups (scissoring)
1570–1600	Stretching vibrations of C=C bonds in the aromatic ring
1690	C=O stretching vibration

## Data Availability

The original contributions presented in this study are included in the article. Further inquiries can be directed to the corresponding authors.

## Abbreviations

The following abbreviations are used in this manuscript:

ATR	attenuated total reflectance
AUC	area under the curve
FTIR	Fourier transform infrared
IQR	Interquartile range
LOD	limit of detection
LOQ	limit of quantification
MSU	Management and Science University
NS	non-smoker
PCA	principal component analysis
PLS-DA	partial least squares discriminant analysis
PRESS	predicted residual sum of squares
ROC	receiver operating characteristic
S	smoker
SPME	Solid Phase Microextraction
ud	undetected
